# US NHANES Data 2013–2016: increased risk of severe obesity in individuals with history of juvenile idiopathic arthritis

**DOI:** 10.1186/s12969-021-00621-2

**Published:** 2021-08-11

**Authors:** Stephanie Merwin, Eleanor Mackey, Sangeeta Sule

**Affiliations:** 1 Division of Psychology and Behavioral Health, Children’s National Hospital, 111 Michigan Ave NW, Washington, DC 20010 USA; 2 Division of Rheumatology, Children’s National Hospital, Washington, DC USA

Dear Editor,

Juvenile idiopathic arthritis (JIA) is the most prevalent rheumatic disease of childhood. Children with JIA have an increased risk of obesity [1, 2], but it is currently unknown whether there are specific differences in risk for development of overweight/Class I obesity or severe obesity (Classes II and III) and whether there are differences in behaviors associated with weight management in adults with a history of JIA.

We used data from the National Health and Nutrition Examination Surveys (NHANES), a program of the National Center for Health Statistics (NCHS) under the Centers for Disease Control and Prevention. NHANES data for the current study included demographic and weight data and Questionnaire data on Medical Conditions from 2013 to 2014 and 2015–2016 [3, 4].

Our sample included 121 respondents with reported history of JIA, diagnosed when the respondent was ≤16 years old. The comparison group was a random sample of respondents without JIA drawn in a 4:1 ratio to the JIA group (*N* = 508). We performed comparison of medians using Mann Whitney Wilcoxon and proportions using Chi-square distribution. Simple and multivariable logistic regression analyses were used to determine the odds ratio (OR) of obesity class.

Those with JIA had an almost three-fold increased odds of Class II (OR: 3, *p* = 0.005) and Class III obesity (OR: 2.7, *p* = 0.002). When controlling for age, gender, and race, respondents with JIA still had an increased odds of Class III obesity (OR: 3.4, *p* = 0.004). Approximately 50% of JIA patients reported trying to lose weight in the past year as compared to 34.5% of those with No Arthritis (*p* = .02; Table 1).

**Table 1:** Respondent demographic information, BMI, and dietary behavior, NHANES data 2013–2014 and 2015–2016

	No Arthritis *N* = 508	Juvenile Idiopathic Arthritis *N* = 121	Juvenile Idiopathic Arthritis vs. No Arthritis *p*-value
Demographic variable			
Current Age, M (SD)	42.6 (1.7)	44.4 (1.5)	0.3
Gender, % female	46.3	53.7	0.2
Race			
% Caucasian	60.2	75	
% African American	13.3	10.6	
% Hispanic	14.7	6.6	
% Other	11.8	7.8	**0.02**
Heaviest self-reported lifetime weight (in kg), M (SD)	84.5 (1.3)	97.6 (2.9)	**< 0.01**
Current BMI, kg/m^2^, M (SD)	27.4 (0.4)	30.6 (0.8)	**< 0.01**
% Overweight or obese, n (%)	339 (66.7)	94 (77.7)	**0.02**
BMI 15–19.9: Underweight, n (%)	29 (5.7)	4 (3.3)	0.3
BMI 20–24.9: Normal, n (%)	140 (27.6)	23 (19)	0.05
BMI 25–29.9: Overweight, n (%)	162 (31.9)	37 (30.6)	0.8
BMI 30–34.9: Class I obesity, n (%)	90 (17.7)	17 (14)	0.3
BMI 35–39.9: Class II obesity, n (%)	28 (5.5)	14 (11.6)	**0.02**
BMI 40 or higher: Class III obesity, n (%)	29 (5.7)	19 (15.7)	**0.0002**
Dietary behavior			
Tried to lose weight in the past year, n (%)	160 (34.5)	46 (50.1)	**0.02**
Changed eating habits, n (%)	72 (14.2)	23 (19)	0.2
Ate less, n (%)	123 (24.2)	43 (35.6)	**0.01**
Skipped meals, n (%)	27 (5.3)	13 (10.7)	**0.02**
Switched to foods with lower calories, n (%)	63 (12.4)	23 (19)	**0.05**
Ate less fat, n (%)	63 (12.4)	26 (21.5)	**< 0.01**
Ate fewer carbohydrates, n (%)	60 (11.8)	26 (21.5)	**< 0.01**
Ate diet foods, n (%)	15 (3)	10 (8.3)	**< 0.01**
Ate more fruits, vegetables, salads, n (%)	89 (17.5)	32 (26.4)	**0.02**
Ate less sugar, candy, sweets, n (%)	69 (13.6)	28 (23.1)	**< 0.01**
Ate less junk food or fast food, n (%)	78 (15.4)	24 (19.8)	**0.2**

*Note*. Bolded *p*-values indicate *p* < .05. NHANES = National Health and Nutrition Examination Surveys. BMI = Body Mass Index. BMI was calculated using height and weight data from the Physical Examination of the NHANES program. Weight was self-reported in pounds and recalculated to kg

History of JIA carries a three-fold increased risk of severe obesity in adulthood, despite increased behavioral efforts to lose weight. Prospective research starting in childhood is needed to identify mechanisms of this relationship and avenues for prevention efforts to avert the development of severe obesity in adulthood. Given adults with obesity are at risk for poor health outcomes including cardiovascular disease, type 2 diabetes, pain, depression and anxiety, and early death [5], this area of research is in critical need of immediate attention. Without prevention efforts aimed at this vulnerable population starting in childhood, the risk of increased morbidity and mortality associated with adult obesity for those with childhood JIA is considerable.

## Data Availability

The datasets generated and/or analysed during the current study are available in the National Health and Nutrition Examination Surveys (NHANES) repository, [https://wwwn.cdc.gov/nchs/nhanes/ContinuousNhanes/Default.aspx?BeginYear=2013] and [https://wwwn.cdc.gov/nchs/nhanes/ContinuousNhanes/Default.aspx?BeginYear=2015].

## Abbreviations

NHANES: National Health and Nutrition Examination Surveys
JIA: Juvenile idiopathic arthritis
BMI: body mass index
NCHS: National Center for Health Statistics
OR: odds ratio

## References

[1] Samad A, Stoll ML, Lavi I, Hsu JJ, Strand V, Robinson TN, Mellins ED, Zisman D, CARRA Legacy Registry Investigators (2018). Adiposity in juvenile psoriatic arthritis. J Rheumatol.

[2] Schenck S, Niewerth M, Sengler C, Trauzeddel R, Thon A, Minden K, Klotsche J (2015). Prevalence of overweight in children and adolescents with juvenile idiopathic arthritis. Scand J Rheumatol.

[3] Centers for Disease Control and Prevention (CDC). National Center for Health Statistics (NCHS). National Health and Nutrition Examination Survey Data. Hyattsville, MD: U.S. Department of Health and Human Services, Centers for Disease Control and Prevention, [2013–2014], [https://wwwn.cdc.gov/nchs/nhanes/ContinuousNhanes/Default.aspx?BeginYear=2013].

[4] Centers for Disease Control and Prevention (CDC). National Center for Health Statistics (NCHS). National Health and Nutrition Examination Survey Data. Hyattsville, MD: U.S. Department of Health and Human Services, Centers for Disease Control and Prevention, [2015–2016], [https://wwwn.cdc.gov/nchs/nhanes/ContinuousNhanes/Default.aspx?BeginYear=2015].

[5] Dixon JB (2010). The effect of obesity on health outcomes. Mol Cell Endocrinol.

